# Superior prognostic accuracy of FIGO staging system in primary female genital tract lymphomas: A retrospective study (IELSG35)

**DOI:** 10.1002/hon.3312

**Published:** 2024-09-26

**Authors:** Maria Cristina Pirosa, Sara Steffanoni, Anna Vanazzi, Fabiana Esposito, Angela Polino, Alberto Schena, Francesco Landi, Maria Elena Cabrera, Saad Akhtar, Santiago Gardella, Osnat Bairey, Astrid Pavlovsky, Anastasios Stathis, Emanuele Zucca

**Affiliations:** ^1^ Oncology Institute of Southern Switzerland Ente Ospedaliero Cantonale Bellinzona Switzerland; ^2^ Institute of Oncology Research Bellinzona Switzerland; ^3^ Valduce Hospital Como Italy; ^4^ Hemato‐Oncology Division, Istituto Europeo di Oncologia Milan Italy; ^5^ Medicine Service, Hematology Section Hospital del Salvador University of Chile Santiago Chile; ^6^ King Faisal Specialist Hospital and Research Centre King Abdullah Center for Oncology and Liver Disease Riyadh Saudi Arabia; ^7^ Institut Catala d'Oncologia Girona Spain; ^8^ Davidoff Cancer Center Rabin Medical Center Petach Tikva Israel; ^9^ Fundaleu‐Fundación Para Combatir la Leucemia Buenos Aires Argentina; ^10^ Centro de Hematologia Pavlovsky Buenos Aires Argentina; ^11^ Faculty of Biomedical Sciences Università della Svizzera Italiana Bellinzona Switzerland

**Keywords:** chemotherapy, CNS prophylaxis, female reproductive system, lymphoma

## Abstract

Primary lymphoma of the female genital tract (PLFGT) is a rare type of extranodal lymphoma. In this retrospective study from the International Extranodal Lymphoma Study Group, we analyzed clinical data from 60 women diagnosed with PLFGT between 1982 and 2012. The median age was 52 years. Limited stage, as defined by the Ann Arbor and FIGO staging systems, was observed in 55% and 63% of cases, respectively. The uterus was the primary site of lymphoma in 25 cases, with the ovaries as the second most common site (*n* = 24). The most common histological subtype was diffuse large B‐cell lymphoma (DLBCL, *n* = 44), followed by follicular lymphoma and marginal zone lymphoma (6 patients each). Two patients received surgery alone as first‐line therapy, while 58 underwent systemic therapy, 16 following major surgery. Thirteen patients received consolidation radiotherapy and six were given central nervous system (CNS) prophylaxis. Twenty patients had disease progression or recurrence. Six patients with DLBCL (14%) experienced CNS relapse, which was the only site of recurrence in five of them. All but one patient with CNS relapse had primary ovarian involvement, and three had bulky disease; none of these patients had received CNS prophylaxis. With a median follow‐up of 60 months, the median overall survival of the DLBCL cohort was approximately 13 years, with a 5‐year survival rate of 77%. In multivariable analysis, advanced disease according to the FIGO system was the only parameter significantly associated with shorter overall, cause‐specific, and progression‐free survival in patients with DLBCL.

## 1 INTRODUCTION

Primary lymphoma of the female genital tract (PLFGT) is a rare condition, accounting for about 1.5% of all non‐Hodgkin lymphomas and 0.5% of all malignant genital tract tumors.^1^ The female genital tract is secondarily involved in 7%–30% of cases of disseminated lymphomas.^2^, ^3^ To date, few series of patients with PLFGT have been published and most of them refer to sporadic cases.^1^, ^2^, ^3^, ^4^, ^5^, ^6^, ^7^, ^8^, ^9^, ^10^, ^11^, ^12^, ^13^, ^14^, ^15^, ^16^, ^17^, ^18^, ^19^, ^20^, ^21^, ^22^, ^23^, ^24^, ^25^, ^26^, ^27^, ^28^, ^29^

PLFGT incidence has increased in the last 40 years, especially in the period from 1997 to 2007.^30^ PLFGT typically affects middle‐aged women over 40 years of age. Although the pathogenesis is poorly known, a protective role of female hormones and menstrual periods has been hypothesized. Furthermore, a possible relationship between chronic inflammation/infection and uterine lymphoma has been reported.^31^, ^32^

Common clinical manifestations include vaginal bleeding, pelvic masses, vaginal discharge, and abdominal pain, which can mimic other genital tract malignancies; systemic B symptoms are quite rare.^33^, ^34^ Occasionally, otherwise asymptomatic PLFGT can be detected through routine gynecologic screening.^2^, ^35^ The uterus and ovaries are the most commonly involved sites in PLFGT,^34^ and diffuse large B cell lymphoma (DLBCL) is the most common histological subtype.^7^

Currently, there is no standard treatment for PLFGT. In the past, localized therapies such as radiotherapy and/or surgery were the primary treatment strategies for early‐stage (stage I‐II) disease. Systemic therapy, often based on the histological subtype, was reserved for advanced‐stage (stage III‐IV) cases. For younger patients, immunochemotherapy alone was recommended for early stages to preserve reproductive function.^2^, ^7^, ^10^ The most common first‐line regimen includes anthracycline‐based therapies (like CHOP regimen: cyclophosphamide/doxorubicin/vincristine/prednisone) with anti‐CD20 monoclonal antibody (rituximab).^16^ However, some studies suggest a conservative approach with local resection followed by adjuvant radiotherapy, which has shown effective long‐term local control.^36^ This approach, however, requires validation with larger cohorts before widespread clinical application.

This retrospective analysis aims to provide an overview of the clinical and histological features, diagnostic approaches, treatment strategies, and outcomes of PLFGT patients observed from 1982 to 2012.

## 2 MATERIALS & METHODS

### 2.1 Patients

IELSG35 is an exploratory multicenter retrospective observational study enrolling patients diagnosed with PLFGT. The main endpoints were: (1) to describe the presenting clinical and histopathologic features, (2) to assess treatment outcomes including response rate, progression‐free survival (PFS), cause‐specific survival (CSS), and overall survival (OS). This study was closed prematurely due to a failure to meet the accrual target. The study was conducted in accordance with the current version of the Declaration of Helsinki and approved by the appropriate Institutional Research Board and Ethic Committee, according to local regulations.

Data on patient characteristics and outcomes were extracted by study investigators from electronic medical records or clinical charts. The extracted information included age, sex, comorbidities (gynecological diseases), lymphoma features (origin site, histology, stage), and biochemical parameters (beta‐2 microglobulin, LDH, hemoglobin, and serology for HIV, hepatitis C and B). Treatment details, along with response rates, relapse rates, and mortality, were also recorded.

The histological diagnosis was based on the WHO 2008 classification.^37^ The primary site was defined as the gynecological site with the largest lesion. The cut‐off size for defining a bulky lesion was 7 cm.

Two separate staging modalities were used: the modified Ann Arbor system, recommended for lymphoma staging by the Lugano Classification,^38^ and the International Federation of Gynecology and Obstetrics (FIGO) system, currently used for gynecological cancers.^39^ Since the latter varies in detail for each gynecological site,^39^, ^40^ we adopted the simpler form proposed for general use in all gynecological lymphomas, with the following definitions: stage I, tumor restricted to the lining and deeper tissue of the involved organ (including bilateral ovarian involvement); stage II, spread by contiguity to immediately contiguous tissues; stage III, involvement of regional lymph nodes; and stage IV, presence of distant involved sites.^2^

In our analysis, we separated patients with localized and advanced disease by dichotomization (stage I‐II vs. stage III‐IV) of either the Ann Arbor or the FIGO system.

### 2.2 Statistical analyses

PFS was calculated from diagnosis until lymphoma progression or death from any cause, CSS until death from lymphoma or its treatment, and OS until death from any cause.^41^ Survival probabilities were estimated using the Kaplan‐Meier method.^42^ Optimal cut‐points for continuous variables were estimated on the receiver operating characteristic curves by the Liu method. The log‐rank test was used for univariable analysis of risk factors (dichotomized age, ECOG performance status, anatomic location, Ann Arbor stage, FIGO stage, bulky disease, LDH levels, international prognostic index (IPI) score, type of frontline therapy). Multivariable analysis, with estimation of hazard ratio and its 95% confidence interval (95% CI), was performed by stepwise backward Cox regression including each factor significantly associated with outcomes at univariable analysis. Differences between the frequencies of categorical data were assessed by the chi‐square test or Fisher exact test as appropriate. A *p*‐value <0.05 was considered statistically significant. Statistical analysis were conducted using the Stata 18.0 software (StataCorp, College Station, TX).

## 3 RESULTS

### 3.1 Study population

The study included 60 women diagnosed with PLFGT between 1982 and 2012 from seven different cancer centers across seven countries (Italy, Spain, Chile, Saudi Arabia, Argentina, Israel and Switzerland). Patient clinical characteristics at presentation, are summarized in Table 1.

**TABLE 1:** Patient's characteristics (*n* = 60).

	*N*	Percent (%)
Age at diagnosis		
≤40 years	15	25
41–55 years	18	30
56–70 years	14	23
>70 years	13	22
Performance status (ECOG)		
0	46	77
1	14	23
Stage (according to FIGO staging)		
localized	38	63
advanced	22	37
Stage (according to Ann Arbor staging)		
I‐II	33	55
III‐IV	27	45
Menopausal status		
Yes	28	47
No	22	37
Unknown	10	16
Bulky disease		
Yes	29	48
No	27	45
Unknown	4	7
Primary gynecological site		
Ovary	24	40
Uterus corpus	12	20
Uterus cervix	13	22
Vagina	10	17
Vulva	1	2
Lymphoma subtype		
DLBCL	44	73
MZL	6	10
FL	6	10
PTCL	2	3
BL	2	3
Symptoms		
*B symptoms*	16	27
*Gynecological symptoms*	18	30
Bleeding	17	28
Vaginal itching	2	3
Vaginal swelling	1	2
*No gynecological symptoms*	15	25
Abdominal pain	10	17
Others	6	10
CNS‐IPI		
Low risk	36	60
Intermediate risk	16	27
High risk	8	13

Abbreviations: BL, Burkitt Lymphoma; CNS‐IPI, central nervous system international prognostic index; DLBCL, diffuse large B‐cell lymphoma; FL, follicular lymphoma; MZL, marginal zone lymphoma; PTCL, peripheral T cell lymphoma.

DLBCL was the most common subtype, occurring in 44 patients, followed by follicular lymphoma (FL) and marginal zone lymphoma (MZL) (6 patients each), Burkitt Lymphoma (BL) (*n* = 2) and peripheral T cell lymphoma (PTCL) (*n* = 2).

In 57 patients the histological diagnosis was obtained by biopsy taken from the involved genital tract organ, in the remaining 3 patients, the diagnostic biopsy was performed in extra‐gynecological tissues (namely, skin infiltrated by contiguity, a regional involved lymph node and the bone marrow, in one patient each).

The median age at diagnosis was 52 years (range: 25–94 years). The most common main presentation sites were the uterus and ovary (approx. 40% each). Forty‐five patients had involvement of a single gynecological organ, while 5 had simultaneous involvement of the ovary and uterus, with lymphoma extending to the vagina in 2 of those cases. Two subjects had concurrent ovarian and vaginal involvement, 5 had involvement of the uterus and vagina, and 1 had involvement of the uterus and vulva. Twenty‐four patients had extra‐gynecological sites involved (7 in bone marrow, 4 in serous membranes, 2 in muscle/skin, 4 in the intestinal tract, 2 in the liver, 2 in the spleen, 1 with involvement of the epidural space and breast, and 4 in the kidney/adrenal glands/bladder). Additionally, 29 (48%) patients had bulky disease.

Moreover, four patients had a previous diagnosis of malignancy: one with localized cutaneous basal cell carcinoma, two with early‐stage colon carcinoma, one with unspecified epithelial carcinoma, and acute myeloid leukemia. Five subjects had autoimmune diseases (rheumatoid arthritis in one case, autoimmune thyroiditis in another, and three cases were unknown), while four had previous gynecological diseases: atrophic vulvovaginitis (*n* = 1), cervical intra‐epithelial neoplasia grade 2 (*n* = 1), condyloma acuminatum (*n* = 1), and one patient underwent fallopian tube resection for an unknown reason.

### 3.2 Treatment outcome

Table 2 describes the initial treatment strategies.

**TABLE 2:** First line treatment (*n* = 60).

First‐line therapy	*N*	%
Surgery alone	2	3
Chemotherapy alone	29	48
Combined therapy	29	48
Surgery and chemotherapy	16	27
Chemotherapy and RT	13	22
Chemotherapy regimen		
CHOP‐Like	17	28
R‐CHOP‐like	30	50
CVP or chlorambucil	3	5
R‐CVP or R‐chlorambucil	3	5
Others	5	8

Abbreviation: CPV, cyclophosphamide/prednisone/vincristine

Two patients underwent surgery alone as first‐line therapy. One, diagnosed with stage II vaginal FL, underwent bilateral hystero‐ovariectomy. The other, diagnosed with extranodal MZL of the uterus, received radical surgery. At a median follow‐up of 10 years, both patients are alive and disease‐free.

Fifty‐eight patients received systemic treatment alone (*n* = 29) or with local therapy (previous surgery (*n* = 16) or previous/subsequent radiotherapy (RT) (*n* = 13)). The median radiation dose administered was 36 Gy (range: 20–40 Gy), with the irradiation field targeting the primary gynecological site in all but one case, where the lumbar/sacral site was irradiated.

Forty‐seven patients received a CHOP‐like regimen, with 30 of them also receiving anti‐CD20 (rituximab). Alkylating‐based regimens (cyclophosphamide/prednisone/vincristine (CVP) or Chlorambucil) were administered in 6 cases, with three of them combined with rituximab. More aggressive sequential regimens (Burkitt‐like regimen) were used in 2 BL cases. Two patients were treated with rituximab alone, and 1 received a vinca alkaloid‐based regimen.

Twenty‐five of 29 (86%) patients treated with (immuno‐)chemotherapy alone achieved a complete response (CR), 1 had a partial response (PR), 1 remained in stable disease and 2 progressed either at the end or during first‐line treatment.

Among the 29 patients treated with combined therapy, 26 responded (23 CR and 3 PR), and 3 experienced disease progression. Specifically, 14 out of 16 patients treated with surgery and chemotherapy responded (13 CR and 1 PR), resulting in an overall response rate (ORR) of 87%, with 2 cases of progression. Additionally, 12 out of 13 patients treated with chemotherapy and radiotherapy responded (10 CR and 2 PR), yielding an ORR of 92%, with only 1 case of progression.

### 3.3 Progression and risk assessment of CNS relapse

Overall, with a median follow‐up of 5 years (IQR, 2.67–7.99), 20 patients experienced relapse or progression. Among two patients treated with surgery alone as first‐line therapy, one relapsed at 18 months from surgery; a second‐line systemic therapy with rituximab and Chlorambucil followed by rituximab of maintenance was performed resulting in CR.

In the subgroup of patients receiving systemic therapy as first‐line treatment, 19 experienced relapse or progression after a median of 7.35 months (range 0.45–86) from the start of chemotherapy. Ten out of these 19 patients received second‐line therapy: 3 with high‐dose cytarabine‐containing regimens, 2 with high‐dose methotrexate‐based regimens for central nervous system (CNS) relapse, and 5 with standard‐dose chemotherapy (including 1 purine analog agent, 1 bendamustine, 1 etoposide, and 2 anthracycline‐based regimens). One patient underwent high‐dose chemotherapy followed by autologous stem cell transplantation (HDC/ASCT) as consolidation treatment, and 2 received radiotherapy consolidation at the relapse site. Among the 10 patients treated at relapse or progression, six died (5 due to progression and 1 due to infection), 1 was lost to follow‐up, and 3 were still alive at the time of database lock. Eight of 9 no‐treated patients at the first relapse/progression moment died after a median time of 25.5 days, one was lost at progression time.

Seven patients (12%) received CNS prophylaxis: five with intravenous high‐dose methotrexate, with or without intrathecal administration, and two with intrathecal methotrexate only. Among these patients, three had a low‐risk CNS‐IPI score^43^ (including the one with epidural BL), three were at intermediate risk, and one was at high risk.

Overall, six patients experienced CNS relapse: five with isolated CNS relapse and one with concurrent CNS and systemic relapse. None of these patients had prior CNS prophylaxis. According to the CNS‐IPI, three were high‐risk, two were low‐risk, and one was intermediate‐risk.

All patients with CNS relapse had DLBCL, with primary ovarian involvement in five and vaginal/uterine involvement in one. Four patients had multiple extranodal sites involved at diagnosis, including renal involvement (two patients) and adrenal involvement (one patient). Three had bulky disease at diagnosis, and 4 had advanced stage. All but one died due to the CNS progression after a median of 26 days from relapse (range 1–1125 days). In comparison with other DLBCL patients, those with CNS relapse had a higher rate of elevated LDH at presentation (80% vs. 21%, *p* = 0.019) and higher frequency of high‐intermediate to high IPI scores (80% vs. 30%, *p* = 0.052).

### 3.4 Survival

After a median follow‐up of 5 years (IQR, 2.67–7.99), 45 patients (75%) were alive, with 43 in CR and 2 with active disease at the time of database lock. Fifteen patients (25%) died: 12 from lymphoma progression, 1 from infection, 1 from colon carcinoma relapse (diagnosed before lymphoma), and 1 from a non‐lymphoma‐related cause. No cases of secondary malignancy were observed.

In the entire cohort, the median OS was over 12 years, and the median PFS was not reached. However, time‐related endpoints varied among histological subtypes, although the differences were not statistically significant (Table 3).

**TABLE 3:** Time‐related endpoints according to histological subtypes (*n* = 60).

Histology (n)	Overall survival		Progression‐free survival		Cause‐specific survival	
	Median (years)	5‐year OS (95% CI)	Median (years)	5‐year PFS (95% CI)	Median (years)	5‐year CSS (95% CI)
DLBCL (44)	10.1	78% (60%–89%)	n.r.	65% (47%–79%)	n.r.	78% (60%–89%)
MZL (6)	n.r.	100%	n.r.	83% (27%–97%)	n.r.	100%
FL (6)	12.7	67% (19%–90%)	1.5	50% (11%–80%)	12.7	67% (19%–90%)
BL (2)	n.r.	100%	n.r.	100%	n.r.	100%
PTCL (2)	4.8	0%	1.7	0%	n.r.	100%

Abbreviations: BL, Burkitt Lymphoma; DLBCL, diffuse large B cell lymphoma; FL, follicular lymphoma; MZL, marginal zone lymphoma; n.r., not reached; PTCL, peripheral T cell lymphoma.

Univariable and multivariable analyses of risk factors associated with outcomes were performed for the largest group of DLBCL patients (*n* = 44). In the univariable analysis, advanced FIGO stage (*p* = 0.0124), elevated LDH (*p* = 0.0465), and high‐intermediate and high‐risk IPI scores (*p* = 0.0061) were significantly associated with poorer OS. Advanced FIGO stage (*p* = 0.0059), elevated LDH (*p* = 0.0300), and high IPI score (*p* = 0.0004) were significantly associated with poorer CSS, along with Ann Arbor stage IV (*p* = 0.0412) and frontline treatment without rituximab (*p* = 0.0361). Advanced FIGO stage (*p* = 0.0050), elevated LDH (*p* = 0.0102), high IPI score (*p* = 0.0038), and treatment without rituximab (*p* = 0.0157) were also associated with shorter PFS. In the multivariable analysis, FIGO stage remained the only statistically significant prognostic factor for OS, CSS, and PFS (Table 4).

**TABLE 4:** Results of multivariable analysis (stepwise Cox regression) in DLBCL subgroup (*n* = 44).

Endpoint	Prognostic factors significantly associated with outcome	HR (95% CI)	*p*‐value
OS	FIGO stage	6.62 (1.20–36.49)	0.030
PFS	FIGO stage	5.66 (1.33–24.09)	0.019
CSS	FIGO stage	12.14 (1.35–108.88)	0.026

Abbreviations: CSS, cause‐specific survival; DLBCL, diffuse large B‐cell lymphoma; OS, overall surviva; PFS, progression‐free survival.

## 4 DISCUSSION

PLFGT is a rare entity, accounting for 0.2%–1.1% of all extranodal lymphomas.^33^ To date, few series of patients with PLFGT have been published, with most reports describing sporadic cases.^1^, ^2^, ^3^, ^4^, ^5^, ^6^, ^7^, ^8^, ^9^, ^10^, ^11^, ^12^, ^13^, ^14^, ^15^, ^16^, ^17^, ^18^, ^19^

Our report on 60 PLFGT cases is one of the largest in the literature. The clinical characteristics of our cohort were similar to those previously reported,^33^, ^44^ and our study has several obvious limitations: it is retrospective, with a limited sample size and includes different histologies. Additionally, the wide time span resulted in remarkable heterogeneity of treatments, including therapies administered in the pre‐rituximab era. Furthermore, all diagnostic biopsy date back over 10 years ago, when molecular subtypes and genomic profile of DLBCL were not assessed as they are today. These factors are now recognized as prognostic and can identify cases at higher risk of CNS relapse.

However, certain findings could impact patient management and merit further discussion.

According to the Ann Arbor system, more than half of the patients (55%) had localized disease (stage I‐IIE), while 45% had disseminated disease. Conversely, according to the FIGO staging, 63% of patients had limited disease and 37% had advanced disease. In the few older studies directly comparing these two staging systems,^1^, ^45^ the Ann Arbor appeared to be a somehow more sensitive prognostic indicator. In our study, however, when the two systems are dichotomized to distinguish between localized and disseminated disease, the advanced gynecological disease defined by the FIGO system was the only significant predictor of shorter OS, CSS, and PFS in multivariable Cox regression models after controlling for all the prognostic factors generated by univariable analysis of each time‐related endpoint.

One could speculate that extranodal involvement, even when the disease is only locally advanced, may lead to a worse prognosis compared to dissemination to distant nodal sites alone. In our study, however, the concurrent involvement of extra‐gynecological sites (such as the intestine, serous membranes and/or urinary tract) often contributed to define an advanced Ann Arbor stage. Hence, it seems appropriate to recommend the integrated use of both staging systems. However, it appears likely that in the near future, functional parameters from PET (such as the total metabolic tumor volume) could be used to enhance the prognostic capability of traditional systems.^46^

Another point worthy of discussion is the risk of CNS relapse, whose incidence is extremely variable in the literature (ranging from 0% to 67%).^47^, ^48^, ^49^ In our cohort it was reported in 6 patients with DLBCL histology (14%). Five of these patients had a primary ovarian disease. This anatomic association was not statistically significant and seems in contrast with prior observations where the uterus involvement was associated ─although not necessarily as *an independent risk factor─* with higher risk of CNS relapse.^48^, ^50^ Even though the recommendation for CNS prophylaxis and CNS relapse risk definition in lymphoma patients are nowadays highly controversial,^51^, ^52^ our results suggest that it may be considered in women with DLBCL of the genital tract with high risk features other such as high CNS IPI or IPI scores.

## 5 CONCLUSION

Despite the several limitations, this is one of the largest retrospective clinical studies in patients with PLFGT, providing valuable insights into the outcomes and natural history of the disease, particularly in terms of CNS recurrence and prognostic factors. Notably, the observation that FIGO staging, typically used for solid tumors, might be more informative than Ann Arbor staging in PLFGT suggests that the use of both staging systems could improve the ability to predict outcomes of these patients. Given the small sample size, these findings need confirmation in larger studies.

## CONFLICT OF INTEREST STATEMENT

MCP: travel grant Beigene, Janssen; EZ: Research Funding (Institutional): AstraZeneca, Beigene, Celgene/BMS, Incyte, Janssen, Roche; Honoraria (Medical educational event): Abbvie; Honoraria (Advisory Board): AbbVie, BeiGene, BMS, Curis, Eli/Lilly, Incyte, Ipsen, Merck, Miltenyi Biomedicine, Roche; Travel grants: AstraZeneca, BeiGene, Janssen, and Gilead; AS: Institutional funding for clinical trials: Abbvie; ADC Therapeutics; Amgen, Astra Zeneca; Bayer; BMS; Cellestia; Incyte, Loxo Oncology; Merck MSD; Novartis; Pfizer; Philogen; Prelude Therapeutics; Roche; Consultant/expert testimony/advisory board (institutional): Debiopharm, Janssen, AstraZeneca, Incyte, Eli Lilly, Novartis, Roche, Loxo Oncology; Travel grant: Incyte; Astra Zeneca; all the other coauthors have not COI.

### PEER REVIEW

The peer review history for this article is available at https://www.webofscience.com/api/gateway/wos/peer-review/10.1002/hon.3312.

## Data Availability

The data that support the findings of this study are available from the corresponding author upon reasonable request.
